# Mapping Intrusive and Non-Intrusive Ultrasound Technologies and Devices Used in Vaginal Examinations During Intrapartum: A Scoping Review

**DOI:** 10.3390/healthcare14152359

**Published:** 2026-08-03

**Authors:** Dereje Bayissa Demissie, Doreen Kainyu Kaura, Kristiaan Schreve

**Affiliations:** 1 Department of Family and Emergency Medicine, Faculty of Medicine and Health Sciences, Stellenbosch University, Stellenbosch 7505, South Africa; 2 Faculty of Community Health Sciences, School of Nursing, University of the Western Cape, Cape Town 7535, South Africa; dkaura@uwc.ac.za; 3 Department of Mechanical and Mechatronic Engineering, Stellenbosch University, Stellenbosch 7600, South Africa; kschreve@sun.ac.za

**Keywords:** automated ultrasound transabdominal, transperineal, intrapartum, ultrasonography, digital VE, cervical dilatation, foetal head station, foetal head position, electrohysterography, labour progress, effacement

## Abstract

**Background**: Technology like ultrasonography (US) has revolutionised the process of measuring cervical dilatation, cervical elasticity, station, and position of the foetal head to assess progress of labour. Ultrasound (US) is deemed non-traumatic, accurate, and user-friendly, offering an objective alternative to traditional vaginal examination (VE). Alternative methods such as position-tracking systems with fingertip sensors have shown limited precision, despite their widespread use. Additionally, US technologies have not been shown to measure effacement, caput, and moulding, as compared to VE. There is a lack of strong evidence supporting US effectiveness in improving outcomes for women and babies. Further research is required to enable respectful care in monitoring of women during labour and birth while eradicating preventable morbidity and mortality by utilising US. **Objective**: This scoping review aimed to map intrusive and non-intrusive ultrasound technologies and devices used in vaginal examinations during intrapartum. **Methods**: This scoping review followed Arksey and O’Malley’s five-step framework and the population, concepts, and contexts (PCC) model. A comprehensive search was conducted across seven databases using refined keywords. The protocol for this scoping review has been registered on the open science framework. The data were extracted, charted, synthesised, and summarised. **Result**: This scoping review included 47 original articles with a combined sample size of over 9000 women in labour or delivery. Most studies focused on ultrasound-based labour monitoring methods such as transabdominal, transperineal, 2D, 3D, and automated approaches—compared to traditional vaginal examination (VE). These ultrasound techniques were consistently praised for their accuracy, reliability, and feasibility in assessing cervical dilation, foetal head station, and angle of progression. Ultrasound was particularly effective in determining foetal head engagement during the second stage of labour, which can influence clinical decision-making and outcomes. Additionally, the review identified emerging intrapartum technologies, including non-invasive purple line observation and low-intensity light imaging probes. These innovations reflect a growing shift toward objective and less invasive labour monitoring methods. Tools like ultrasound and automated tracking algorithms demonstrated superior sensitivity, specificity, and patient comfort compared to VE. However, challenges remain in clinical applicability, standardisation, and implementation, particularly in resource-limited settings. Overall, the findings suggest a transition toward digital and ultrasound-based technologies as central components of modern labour monitoring, with further research needed to validate newer devices and ensure equitable integration into clinical practice. **Conclusions**: This scoping review highlights a shift toward ultrasound-based technologies transabdominal, transperineal with 2D/3D, and automated as more accurate and patient-friendly alternatives to vaginal examination. These methods improve assessment of cervical dilation, foetal head station, and progression angle. Emerging tools like the purple line and light imaging probes show promise but require further validation. Policymakers should support investment in affordable digital tools; clinical practice must prioritise training and integration; and research should focus on standardisation, long-term outcomes, and feasibility to ensure equitable and effective implementation of these technologies in labour monitoring.

## 1. Introduction

This scoping review explores global evidence on digital technologies used during the intrapartum period, mapping intrusive and non-intrusive ultrasound devices for vaginal examinations to monitor labour progress in women.

“Intrusive” methods in intrapartum care are those involving physical penetration or internal examination, such as vaginal examination (VE). VE is widely used to assess cervical dilation and foetal position but is considered intrusive due to its invasive nature, potential for discomfort, psychological impact, and increased risk of infection [1]. In contrast, “non-intrusive” methods refer to techniques that do not involve internal access to the body. Transabdominal ultrasound, excluding transvaginal types, is considered non-intrusive. It enables accurate foetal position assessment, reduces patient discomfort and infection risk, and supports continuous digital monitoring, enhancing clinical decision-making and improving the safety and efficiency of intrapartum care [1].

Previous systematic reviews identified that the traditional vaginal examination (VE) is subjective and potentially inaccurate in monitoring the progress of labour [2]. While routine VEs are widely used, there is limited evidence supporting their effectiveness in improving outcomes for women and babies [3]. Ultrasound technology has emerged as a valuable tool in obstetric care, offering promising capabilities for predicting the likelihood of successful vaginal delivery and supporting safer operative interventions [4]. In particular, intrapartum ultrasound has shown greater accuracy in monitoring labour progression compared to traditional vaginal examinations [4].

The study by Hassan et al., 2014 [5], introduced sonopartograph as a new ultrasound-based method called the “sonopartogram” for assessing labour progress, including cervical dilatation and foetal head descent and rotation. The agreement between VE and ultrasound was good for cervical dilatation and head rotation, but less so for head descent.

A systematic review and meta-analysis found that vaginal examination is essential for data collection for populating the partogram. Further, transperineal ultrasound can be used as an auxiliary tool to VE. However, publication bias in ultrasound parameters may overestimate diagnostic performance [1]. Non-invasive methods for sonographic imaging parameters can also be used, but vaginal examination remains the standard. This study highlights the need for more accurate methods for evaluating labour progress during the first stage [1].

A study conducted in Türkiye [6] focused on developing an AI-assisted device for electronic vaginal examinations and monitoring labour progress. The research led to the creation of an innovative product that is reported to be safe for both groups. This device, not yet used in Türkiye or globally, enables electronic labour follow-up in obstetrics, supports patient and employee safety, and maintains labour follow-up effectiveness. It digitises the partograph, allows simultaneous patient recording, and provides a decision support system to detect abnormalities during labour, enhancing the safety, efficiency, and quality of labour monitoring [6].

A systematic review and meta-analysis conducted by Pan et al. (2022) [1] found that transperineal ultrasound moderately predicts labour progress using parameters like head–perineum distance (HPD) and angle of progression (AOP). HPD showed a pooled sensitivity of 0.74 and specificity of 0.77. Despite promising results, the overall evidence quality was low, highlighting the need for more rigorous studies to confirm diagnostic accuracy. Despite promising findings, further research is essential to refine ultrasound techniques for measuring cervical dilation [2]. Future studies should prioritise developing accurate, non-invasive tools for labour assessment, while incorporating women’s perspectives and preferences [3]. Advancing objective measurement methods remains a key goal in improving obstetric care. Vaginal examination (VE) remains the standard method for assessing labour progress; however, it is subjective, invasive, and often uncomfortable for women, with variability in accuracy depending on the examiner’s experience [3]. These limitations underscore the need for more objective, consistent, and patient-friendly alternatives.

Ultrasound technologies, particularly non-intrusive methods like transperineal and intrapartum ultrasound, have shown promise in providing accurate, real-time assessments of labour progression. Parameters such as head–perineum distance (HPD) and angle of progression (AOP) offer quantifiable insights, yet the diagnostic performance and clinical integration of these tools remain inconsistent across studies [1,4]. Moreover, the evidence base is fragmented, with varying methodologies and devices used in practice. Given this context, a scoping review is warranted to systematically map the landscape of both intrusive and non-intrusive ultrasound technologies and devices currently employed during intrapartum care. This review aims to identify gaps, consolidate existing knowledge, and guide future research toward developing reliable, non-invasive tools that align with clinical needs and respect women’s preferences. This scoping review, guided by the population, concept, and context (PCC) framework, aims to map globally available evidence on digital technologies, both intrusive and non-intrusive, used in vaginal examinations during intrapartum care for women aged 15–49, focusing on ultrasound-based and other digital technologies for monitoring labour progress.

## 2. Review Questions

The present scoping review aims to answer the following questions:

1. What intrusive and non-intrusive prototypes are used for monitoring the progress of labour by measurement?
2. What ultrasound-based digital health or mobile application devices are developed for monitoring progress of labour by measurement (cervical dilatation, cervical effacement, descent of the head, moulding, and caput)?
3. What is the accuracy of existing developed artefacts and devices used to monitor the progress of labour?

## 3. Methods

This scoping review was carried out following the method suggested by Arksey and O’Malley [7,8]. The following five steps were used: Step 1: Identifying the research question; Step 2: Searching relevant studies; Step 3: Selecting appropriate studies; Step 4: Charting the data; and Step 5: Collating, summarising, and reporting the results. The optional Step 6, consultation with relevant stakeholders, has been excluded from this review. The incorporation of recommendations made by Levac and colleagues was considered [9]. A scoping study approach enables systematic searching, selecting, and examining of the literature; knowledge synthesis; and mapping of evidence to address research questions [7,8]. The Preferred Reporting Items for Systematic Reviews extension for Scoping Reviews (PRISMA-ScR) proposed was used to present the scoping review report [9]. The PRISMA-ScR provides a reporting guideline containing 20 essential items and 2 optional items included in scoping reviews [9]. This guideline also facilitates methodological transparency and acceptance of the research findings [9]. The protocol for this scoping review has been registered on the Open Science Frameworks https://osf.io/b6ujr/ (accessed on 13 February 2025).

- Step 1. Identifying the research question: The present scoping review aims to answer the following questions:
  1. What intrusive and non-intrusive prototypes are used for monitoring the progress of labour by measurement (cervical dilatation, cervical effacement, descent of the head, identifying moulding and caput)?
  2. What ultrasound-based digital health or mobile application devices are developed for monitoring progress of labour by measurement (cervical dilatation, cervical effacement, descent of the head, identifying moulding and caput)?
  3. What is the accuracy of existing developed artefacts and devices used to monitor the progress of labour (cervical dilatation, cervical effacement, descent of the head, moulding, and caput)? The research questions were identified using the people, concept, and context framework approach (Table 1).

- Step 2. Identifying relevant studies
  1. Search strategy

The search strategy aim was to locate both published and unpublished studies. Five databases were systematically searched, along with the grey literature, and data were extracted, charted, synthesised, and summarised. The search strategy involved a brief initial search of Medline and CINAHL, followed by keywords from the titles and abstracts of articles found in Cochrane Review Library, PubMed, CINAHL, Medline, EMBASE, Scopus, and Web of Science. The scoping review included the grey literature searches. The search involved formulating research questions; identifying relevant studies; selecting eligible studies; charting data; and collating, summarising, and reporting the results. This study also included grey literature searches. The text words contained in the titles and abstracts of relevant articles, and the index terms used to describe the articles were used to develop a full search strategy to report the name of the relevant databases/information sources (see Appendix A). A complete search strategy that employed medical subject headings (MeSH) and keywords was used. The Boolean terms (AND and OR) were used to separate keywords, and Medical Subject Headings terms (MeSH) were used in the advanced search of articles; see details in the Supplementary Files. The search strategy, including all identified keywords and index terms, was adapted for each included database and/or information source. The reference list of all included sources of evidence was screened for additional studies by using a snowball approach; a secondary search of relevant articles from reference lists of included studies was undertaken in the systematic review, or references of systematic reviews on the same or similar topic. Studies published in the English language and published from 1 January 2000 to 14 February 2025 were included. The rational justification for the selection of the year 2000 as the starting point was based on the emergence of intrapartum ultrasound applications in the clinical literature around that time. See details in Search supplementary file1.

  2. Study selection: Selecting eligible studies

The study eligibility criteria were based on the Joanna Briggs Institute’s PCC framework [7,9]. The study types included ‘primary studies published in peer-reviewed journals accessible online and through interlibrary requests’. Non-English-language articles were excluded. Eligible articles were uploaded into EndNote 21 reference management software [10], and duplicates were removed. Title and abstract screening and full-text reviews were performed independently by two researchers (DBD, DKK), with a third reviewer if significant discrepancies could not be resolved.

- Step 3. Study selection

Two review authors independently evaluated the titles and abstracts of the included articles. Full-text articles were obtained for all titles and abstracts that met the eligibility criteria, and two authors evaluated the ‘full-text publications independently to determine which studies should be included in the review based on eligibility criteria’.

**Table 1:** PCC (people, concept, and context) used to identify review questions.

People	The population included in this review comprised women of reproductive age (15–49 years), specifically those who were pregnant, in labour, or undergoing delivery
Concept	transperineal (ultraso* OR sonog*) AND clinical examination in labour OR transperineal (ultraso* OR sonog*) AND digital examination in labour or transabdominal (ultraso* OR sonog*) AND clinical examination in labour or transabdominal (ultraso* OR sonog*) AND digital examination in labour or Intrapartum (ultraso* OR sonog*) OR Ultrasound (4D Transperineal Ultrasound,3D/2D Ultrasound AND rotation or Intrapartum (ultraso* OR sonog*) AND position Obstetric Examination
Context	All available evidence were mapped globally ► Published from 1 January 2000 to 15 February/2025 ► Written in English ► All primary studies (quantitative, qualitative, and mixed-method published articles) – Quantitative (cross-sectional/observational) – Qualitative (phenomenology, case study) – Mixed-method research published articles

### 3.1. Eligibility Criteria

This ‘scoping review’ included articles based on specific eligibility criteria:

1. Studies involving pregnant women for labour/delivery follow-up/labour progress/birth/childbirth;
2. Studies that reported evidence of digital devices/technologies used to measure vaginal examination and accurately estimate the progress of labour;
3. Studies from any part of the world;
4. All study designs (interventional, observational, mixed study, quantitative, and qualitative);
5. English-language publications.

This scoping review focuses on studies published between 2000 and 2025 to gather recent evidence on digital devices used for measuring vaginal examination and accurately estimating labour progress.

### 3.2. Studies Excluded from This Scoping Review

The review team excluded articles published in languages other than English due to constraints related to time, funding, and the lack of necessary language skills and resources to handle non-English studies and access to specialised databases.

Studies that focused on routine vaginal examinations other than digitalized partogram or ultrasound, or non-intrusive or digital cervimetry, were excluded.

### 3.3. Study/Source of Selected Evidence

**Figure 1:**
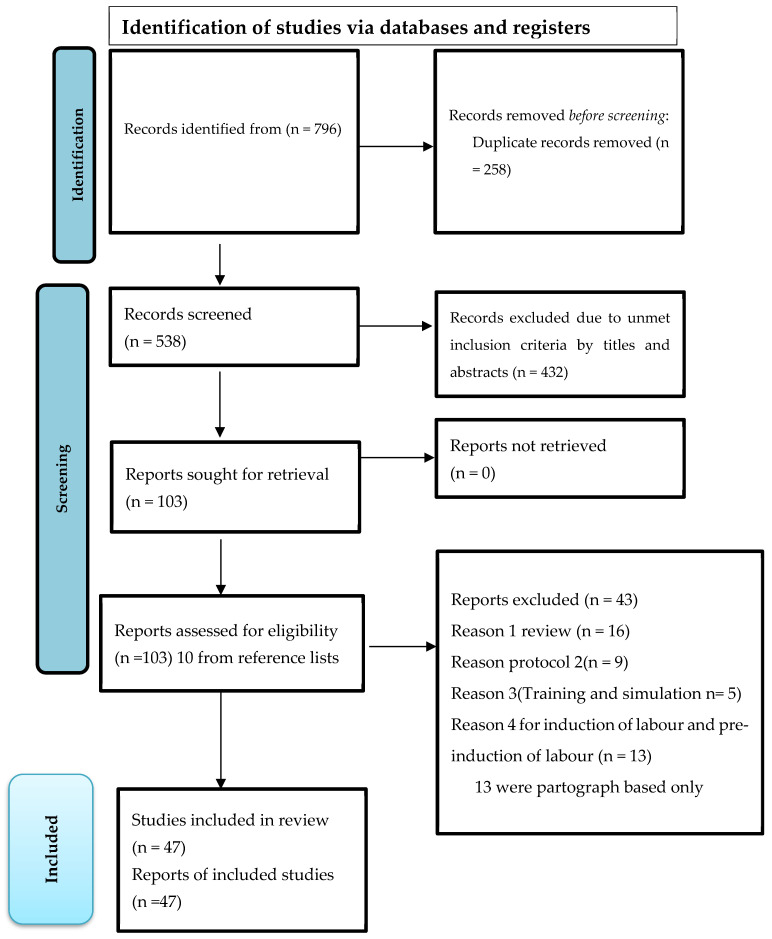
PRISMA-ScR 2020 flow diagram for scoping reviews, which included searches of databases, registers, and other sources [11].

The three review authors conducted a thorough title screening using electronic databases guided by eligibility criteria. All relevant articles were imported into an EndNote library software for duplication and were shared with the review team for the next stage of the study screening and selection process. Duplicate records were checked and removed. A screening process for the abstract and full-text screening phases was developed using eligibility criteria. Three review authors independently completed the abstract and full-text screening phases and collected data to include categories. Discrepancies in abstract screening were addressed through discussion by the review team until a consensus was reached. The PRISMA-ScR flow diagram was used to report the screening results [11] (Figure 1). The full texts of the selected citations were assessed in detail against the inclusion criteria by three independent reviewers. The reasons for exclusion of full-text sources of evidence that did not meet the inclusion criteria are recorded and reported in the scoping review. Any disagreements that arose between the reviewers at each stage of the selection process resolved through discussion, or with an additional reviewer/s. The results of the search and the study inclusion process will be reported in full in the final scoping review and presented in a PRISMA-ScR flow diagram. Three reviewers independently screened the titles and abstracts of 796 articles, resolving disagreements on four items through discussion. Based on this initial screening, 141 articles were selected for full-text review. An additional 10 relevant articles were identified through reference list searches, bringing the total number of articles retrieved for full-text assessment to 151. After applying the inclusion criteria, 47 articles were found to be eligible and are included in the current scoping review [12,13,14,15,16,17,18,19,20,21,22,23,24,25,26,27,28,29,30,31,32,33,34,35,36,37,38,39,40,41,42,43,44,45,46,47,48,49,50,51,52,53,54,55,56,57,58].

- Step 4. Charting the data
  1. Data extraction

All relevant data from the included articles were extracted after two authors (DB, DKK) thoroughly read the full text, and the extracted study characteristics were used to test and refine the data extraction tool before its use using 10 eligible studies, including study authors, title, country/setting, aim/purpose of the study, sample size, study design, population, aim(s) or research question, data source, data collection methods, data analysis, participant characteristics, key findings and limitations, and type of digital devices/technologies addressed, with findings that were relevant to conclusions and recommendations regarding vaginal examination by ultrasound (4D transperineal ultrasound, 3D ultrasound, the purple line, low-intensity light imaging probe for obstetric examination). Data were extracted from papers included in the scoping review by two independent reviewers using a spreadsheet of key factors that were developed to extract relevant data from each included study.

- Step 5. Collating, summarising, and reporting the results

According to Arksey and O’Malley [8], scoping reviews require thematic frameworks to present narrative accounts of the selected literature. The data were grouped thematically by reading and rereading the data items and grouping similar/related items into iteratively developed themes. A PRISMA-ScR flow diagram was used to demonstrate the study selection process and search results [7,9,11]. A descriptive numerical summary was used to present the characteristics of the included studies. Qualitative thematic synthesis was also conducted using a data-driven bottom-up approach. The results were classified under the main conceptual categories, such as ‘ultrasound-based technologies used in vaginal examination, the non-intrusive purple line, and low-intensity light imaging probes’. Under each category, we further provided data on the article’s characteristics, including but not limited to the total number of studies, types of study design, sources of data, years of publication, and key findings (**see Appendix A and Data extraction instrument.xlsx file 2**). Tables and figures were used to present the results in line with the aims of the scope of the review. The implications of the results were elaborated upon considering research and practice to help us identify gaps in vaginal examinations and to develop a non-intrusive AI-enabled prototype for vaginal examinations, integrating AI algorithms to analyse and interpret data, ensuring accuracy and minimising patient discomfort.

- Step 6. Methodological quality appraisal

JBI quality appraisal tools were used to assess the methodological quality of the research publications by evaluating the extent to which they addressed the possibility of bias in areas of study design, conduct, and analysis [59,60,61] Supplementary (PRISMA-ScR) Checklist file 3).

Two researchers (D.B.D., D.K.K.) independently assessed each included paper, and any uncertainties regarding the quality of publications were resolved through discussion. Although a formal assessment of the methodological quality of the included studies was performed, articles with poor quality were not excluded in scoping reviews, and this step was excluded in the current scoping review.

## 4. Results

### 4.1. Study Characteristics

A total of 47 original articles with a total sample size of 9000 women in labour or delivery were included in this scoping review [12,13,14,15,16,17,18,19,20,21,22,23,24,25,26,27,28,29,30,31,32,33,34,35,36,37,38,39,40,41,42,43,44,45,46,47,48,49,50,51,52,53,54,55,56,57,58], where the studies were conducted across a variety of geographical locations, including Europe (UK, France, Norway, Italy), Asia (India, China, South Korea), Africa (Ghana, Kenya, Nigeria, Egypt), South America (Brazil), the Middle East (Israel, Türkiye, Iran), and North America (USA). This provides a broad perspective on labour management practices in different healthcare systems.

The studies involved a diverse group of pregnant women undergoing labour, including those with both low-risk and single pregnancies. The sample sizes varied considerably, ranging from a small pilot study with around 25 participants in Belgium [28] to larger cohort studies with several hundred women (up to 2880 in the case of the study in Nigeria [56]).

Most of the studies employed prospective observational cohort designs, which allowed for the systematic collection of data on labour progress using both traditional methods, like VE, and newer techniques (ultrasound). See details in Supplementary data set .xlsx file 4.

Table 2 shows that in summary, the research landscape on vaginal examination and labour follow-up is characterised by studies with diverse settings, participants, and study designs, reflecting the growing interest in improving labour management practices worldwide; see the details in Table 2. This table and summary provide a concise overview of the research landscape regarding digital devices and technologies for labour monitoring.

### 4.2. Ultrasound-Based Technologies Used in Monitoring the Progress of Labour and Birth

This report details ultrasound technology types, sample sizes, and study designs across 40 studies. The majority of the forty (40) studies with 3416 sampled women during birth with a focus on labour monitoring focus on ultrasound-based methods. These include transabdominal, transperineal, 2D, 3D, and automated approaches, compared to traditional VE [12,13,14,15,16,17,18,19,20,21,22,23,24,25,26,27,28,29,30,31,32,33,34,35,36,37,38,39,40,41,42,43,44,45,46,47,48,56,57].

In the 40 total studies of ultrasound-based technologies used in monitoring the progress of labour and birth and labour follow-up, the ultrasound technology types used are highly diversified from transperineal ultrasound (TPUS), with different dimensions, from 2D to 4D, and other types (intrapartum ultrasound, automatic sonographic measurement, wireless mobile, transvaginal, and transabdominal) also applied for labour monitoring and birth follow-up.

The sample sizes of study participants ranged from 10 in the intrapartum echographic monitoring in Italy [16] to 613 participants in the novel ultrasound partogram in France [24]. Also the devolvement of the proof of concept was not mentioned for one study in this report.

Almost more than 98% were prospective observational studies and comparative studies with routine vaginal examination.

These technologies measure various parameters, such as cervical length, dilation, and position; foetal head descent and rotation; angle of progression (AOP); head–perineum distance (HPD); and head–symphysis distance (HSD), as the major included points of measurement. See details in Table 2.

### 4.3. Key Findings on Non-Invasive Methods (Ultrasound-Based Technologies) Compared to Invasive Methods (Digital Vaginal Examination)

Ultrasound-based technologies offer precise, non-invasive imaging for labour monitoring, enhancing accuracy in determining foetal position. They improve patient comfort by reducing discomfort and psychological impacts, minimise infection risks, and digitise labour progress for continuous recording and decision support, ensuring efficient and safe labour monitoring [1].

Vaginal examination (VE) is the traditional standard for assessing labour progress, especially cervical dilatation. VE is uncomfortable and carries infection risks but remains crucial for clinical decisions like assessing cervical dilatation and foetal position [1].

This study found that ‘vaginal examination (VE)’ and ultrasound have similar data completeness, but neither technique has superiority. DVE is poor at determining foetal head position, with its low accuracy not improving with increasing cervical dilatation. Ultrasound is useful in early labour but less effective in active labour. DVE is subjective and affected by caput in the head station [41].

This study found a high error rate of 76% in transvaginal digital foetal head position determination during active labour using ultrasound assessments. Attending physicians had a two-fold higher success rate in determining correct foetal head position compared to residents in the ±45° analysis. Intrapartum ultrasound increases the accuracy of foetal head position assessment during active labour and may serve as an educational tool for physicians in training [38].

In general, ultrasound-based methods are praised for their accuracy, reliability, and feasibility in measuring parameters like cervical dilation, foetal head station, and angle of progression. Several studies highlight the benefits of ultrasound in determining the foetal head engagement in the second stage of labour, which could determine the decision regarding labour and labour outcomes.

Ultrasound is a crucial tool for assessing labour progression, providing non-invasive measurements of cervical dilation, foetal head station, and angle of progression. Key applications include transabdominal and transperineal ultrasound, which measure head–perineum distance, angle of progression (AoP), and foetal head descent [31,47]. It is a reliable alternative to Ves, with high interobserver agreement [12,15,31,47]. Three-dimensional and automated ultrasound systems provide consistent and accurate readings for angle of progression (AoP) and the head–perineum distance (HPD) [13,34]. The sonopartogram combines ultrasound with the partogram concept to track cervical dilation and foetal head position [5].

These review results have demonstrated that automated measurement of the ‘head–perineum distance (HPD)’ during labour is feasible and correlates well with gold-standard manual measurements performed by senior experts. This newly developed algorithm has the potential to assist clinicians in performing intrapartum ultrasound, which has recently been suggested to enhance labour management [12]. Implementation challenges include training and standardisation of ultrasound techniques, the potential increase in examination time and resources, and variable availability of ultrasound equipment across healthcare settings. However, further research is needed to standardise techniques, assess long-term outcomes, and determine the optimal integration of these technologies into routine clinical practice. See details in Table 3.

### 4.4. Other Technologies (Purple Line, Low-Intensity Light, and Other Instruments) Used in Monitoring the Progress of Labour and Birth Follow-Up

The “purple line” assessment in labour is a non-intrusive method to assess labour progress, offering an alternative to vaginal examinations by observing a line of discoloration in the sacral region [54]. However, the position is not comfortable for pregnant women, similarly to intrusive vaginal examinations. Using a low-intensity light imaging probe is a non-intrusive method for assessing labour progress; it allows for objective visualisation of the cervix without physical insertion, unlike vaginal examinations (VEs) [55].

The recently emerging technologies for labour progress estimation include the purple line, low-intensity light imaging probes, disposable instruments, and Dila Check (interexaminer agreement). In seven studies with 3986 women in labour, the technologies used include visualising cervical dilation, the purple line method, non-invasive purple line observation, and low-intensity light imaging probes. The non-invasive purple line observation and low-intensity light imaging probes are used for vaginal examinations in various settings, including India, Bangladesh, Egypt, Brazil, Scotland, Iran, Serbia and Montenegro, Nigeria, and Australia, and some invasive examinations are also used, like simple instruments for measuring cervical dilatation during labour with disposable instruments, and Dila Check (interexaminer agreement) [51,52]. The study designs include prospective crossover studies, observational cohort studies, comparative cross-sectional studies, interventional studies, randomised comparative studies, quasi-experimental studies, quality improvement projects, cross-sectional studies, hospital-based prospective analytical studies, longitudinal studies, non-equivalent control group post-test-only designs, and laboratory-based investigations [49,50,51,52,53,54,55,61]. The purple line is a diagnostic tool for cervical dilatation and foetal head station, while cervical dilation measurement devices include the Dila Check and low-intensity light imaging probe. These studies suggest a shift towards more objective and potentially less invasive labour monitoring methods, with digital and ultrasound technologies playing a central role; see details in Table 4.

### 4.5. Key Findings on Other Emerging Technologies and Novel Prototypes Used in Monitoring the Progress of Labour and Birth Follow-Up

Other technologies and novel prototypes are used, including emerging technology aiming to address limitations of traditional methods, like a prototype device for visualising cervical dilation and reducing subjectivity associated with manual examinations. The purple line method is a visual observation of the purple line as a proxy for cervical dilation and foetal head station. Finally, there are some studies on the non-invasive purple line observation which has the potential to supplement the evaluation of labour progression, and some studies on novel prototypes like a low-intensity light imaging probe. Novel devices like low-intensity light imaging probes are explored, though they often have limited clinical validation at this stage [49,50,51,52,53,54,55,61]. This analysis provides a snapshot of the research focus regarding labour monitoring technologies during this study period. It highlights the growing interest in interventional studies, the continued importance of ultrasound, and the emergence of innovative but less-validated devices. Overall, the studies indicate a move towards more objective and potentially less invasive methods of labour monitoring, with digital and ultrasound technologies playing a central role. However, feasibility, cost, and the need for training remain important considerations for widespread implementation; see details in Table 5.

### 4.6. Accuracy of Existing Developed Artefacts and Devices Used to Monitor the Progress of Labour (Cervical Dilatation, Cervical Effacement, Descent of the Head)

The report compared traditional vaginal examination (VE) and ultrasound methods for labour assessment outcomes. Ultrasound showed high agreement for cervical dilation and higher accuracy for foetal head position compared to standard practice (VE). However, VE did not measure angle of progression and head–perineum distance. Ultrasound showed high sensitivity and specificity for predicting delivery mode for both measures, with significant differences. All comparisons in multiple studies show that ultrasound methods offer improved patient experience compared to traditional VE, with less discomfort and pain, higher patient preference, and reduced need for invasive examinations [5,12,13,14,15,16,31,33,34,35,36,37,38,39,40,41,42,43,44,45,46,47,48]. The evidence suggests that ultrasound-based examination technologies offer the potential for improving the accuracy of labour monitoring while enhancing patient comfort and potentially reducing complications.

The accuracy of foetal head rotation assessment was significantly higher with ultrasound (98%) compared to vaginal examination (VE), which achieved only 46% accuracy, representing a statistically significant difference (*p* < 0.001) [5]. The mean difference in rotation measurements was −3.9°, with wide limits of agreement, indicating variability in VEs and reinforcing the reliability of sonographic methods.

The 3D automated ultrasound measurements were feasible in all cases and acceptable in 90.4% of initial assessments, reaching 100% after repeats. The 3D automated technique demonstrated good intra- and interobserver reproducibility and showed very good agreement with the manual technique. However, the 3D automated measurements were significantly wider than manual measurements (133 ± 17° vs. 118 ± 21°, *p* = 0.013). While the assessment of the angle of progression (AoP) through 3D ultrasound was highly reproducible, the automated software systematically overestimated AoP compared to the manual technique, limiting its current clinical applicability [34].

In the birth simulator, the automatic identification of the symphysis distal end and foetal head outermost point was correct in 98% of images, providing high visual reliability. The algorithm achieved real-time monitoring through maximisation of the similarity coefficient. It provided a global accuracy of 0.9 ± 4.0 mm for FHS and 4° ± 9° for PA, significantly surpassing the routine method’s diagnostic ability, making it a reliable tool for early identification of abnormal labour patterns and supporting clinical decisions [16].

The median cervical dilation measured by transperineal ultrasound (TPUS) was 5.1 cm, compared to 5 cm by cervical examination (CE), with a strong positive correlation (r = 0.86 (95% CI 0.72 to 0.93); *p* < 0.001). TPUS was significantly less painful than CE, with median pain scores of 0 vs. 2, respectively (*p* < 0.001). All participants preferred TPUS over CE. Conclusions: TPUS provides a non-invasive, convenient alternative to traditional digital exams for measuring cervical dilation in term labouring patients, with strong correlation with CE [18].

This study found a strong correlation between the automatic sonographic and manual methods for measuring delta-HPD (ICC = 0.97) and delta-AoP (ICC = 0.99). The automatic algorithm demonstrated high accuracy, with a coefficient of determination (r^2^) of 0.98 for both measurements and low residual errors (1.2 mm for delta-HPD and 1.5° for delta-AoP). Bland–Altman analysis showed mean differences of 0.52 mm for delta-HPD and 0.35° for delta-AoP between the two methods, indicating good agreement [13].

This review’s results have demonstrated that automated measurement of the head–perineum distance (HPD) during labour is feasible and correlates well with gold-standard manual measurements performed by senior experts. This newly developed algorithm has the potential to assist clinicians in performing intrapartum ultrasound, which has recently been suggested to enhance labour management [12]. Implementation challenges include training and standardisation of ultrasound techniques, the potential increase in examination time and resources, and variable availability of ultrasound equipment across healthcare settings. However, further research is needed to standardise techniques, assess long-term outcomes, and determine the optimal integration of these technologies into routine clinical practice.

Critics note that none of the identified scoping studies have reported accuracy in monitoring cervical effacement, moulding, and caput. These limitations of the existing technologies indicate the need for further research for these methods to fully replace intrusive vaginal examinations.

## 5. Discussion

This scoping review highlighted that the evidence mapping of existing technologies for examining the progress of labour and birth found that VE is poor at determining foetal head position, is subjective, is affected by caput in foetal head station, and results in low accuracy. Furthermore, ultrasound was found to be useful in early labour but less effective in active labour [41]. This evidence, supported by a review of the literature on intrapartum sonographic assessment of labour, highlights the growing importance of intrapartum ultrasound, which is useful not only for determining the foetal head position before applying a vacuum in the obstructed second stage of labour, but also for enabling the assessment of labour progression and aiding skilled birth professionals in making decisions about assisted delivery [62]. This implies that soon, intrapartum sonography will become an essential tool for managing labour, enhancing the quality of care provided to patients.

This study revealed a high error rate of 76% in determining foetal head position during active labour using transvaginal digital methods. Attending physicians were twice as successful as residents in correctly identifying the foetal head position within a ±45° range. Intrapartum ultrasound significantly improved the accuracy of foetal head position assessment and could be a valuable educational tool for training physicians [38]. A review conducted by Kido et al. found that transperineal ultrasonography, integrated into intrapartum ultrasound, enhances auxiliary diagnosis and is a key point-of-care tool in labour wards in addition to aiding in observing postpartum pelvic floor muscles and managing lower urinary tract symptoms, making it a versatile tool for perinatal management [63].

In general, ultrasound-based methods are praised for their accuracy, reliability, and feasibility in measuring parameters like cervical dilation, foetal head station, and angle of progression. Several studies highlight the benefit of ultrasound to determine the foetal head engagement in the second stage of labour, which could determine the decision about labour and the labour outcome. This scoping review’s findings, supported by a previous systematic review and meta-analysis performed by Pan et al., confirms that transperineal ultrasound is promising for determining head–perineum distance (HPD) and angle of progression (AOP) parameters, reducing the need for vaginal examinations in the first stage of labour [1].

Ultrasound emerged as crucial tool for assessing labour progression, providing non-invasive measurements of cervical dilation, foetal head station, and angle of progression, and its identified key applications include transabdominal and transperineal ultrasound, which measure head–perineum distance, AoP, and foetal head descent [31,47]. It is a reliable alternative to VE with high interobserver agreement [12,15,31,47]. These findings are supported by a previous systematic review and meta-analysis which found that transperineal ultrasound is promising for determining head–perineum distance (HPD) and angle of progression (AOP) parameters, reducing the need for vaginal examinations in the first stage of labour [1].

The recent technologies used, like 3D and automated ultrasound systems, provide consistent and accurate readings for angle of progression (AoP) and the head–perineum distance (HPD) [13,34]. All comparisons in multiple studies show that ultrasound methods offer improved patient experience compared to traditional digital vaginal examination. US results in less discomfort and pain, higher patient preference, and reduced need for invasive examinations [5,12,13,14,15,16,31,32,33,34,35,36,37,38,39,40,41,42,43,44,45,46,47,48]. These findings are strongly supported by a systematic review and meta-analysis performed by Wiafe. et al., which found that intrapartum ultrasonography is more effective than DVE in assessing foetal head position during the first stage of labour, with a significantly higher success rate for ultrasound [64].

The birth simulator algorithm accurately identifies key landmarks, enabling real-time monitoring and outperforming routine methods in diagnosing abnormal labour patterns with high precision [16].

The accuracy of the foetal head rotation assessment was significantly higher with ultrasound (98%) compared to vaginal examination (VE), which achieved only 46% accuracy, representing a statistically significant difference (*p* < 0.001) [5]. The mean difference in rotation measurements was −3.9°, with wide limits of agreement, indicating variability in VEs and reinforcing the reliability of sonographic methods. This finding highlights the superior precision of the sonopartogram, which enables objective and reproducible data collection during labour. This result stands in contrast to findings from universal transvaginal ultrasound cervical length screening programmes in low-risk singleton pregnancies. Despite targeted treatment for women with a cervical length ≤25 mm, these programmes did not significantly reduce preterm birth rates [65]. Moreover, the incidence of a cervical length ≤20 mm was only 1.1%, and protocol deviations occurred in 43% of cases. The rate of spontaneous preterm birth remained similar between screened and unscreened groups, suggesting limited clinical impact in this context [66]. Taken together, these findings highlight a critical distinction: ultrasound technologies are more effective in intrapartum monitoring than in antenatal screening for preterm birth in low-risk populations. The high accuracy of sonographic tools during active labour, particularly for foetal head rotation, station, and progression angle, supports their integration into routine intrapartum care. In contrast, the limited predictive value and the implementation challenges of cervical length screening programmes underscore the need for more targeted approaches and improved adherence to protocols. These insights reinforce the importance of context-specific application of ultrasound technologies. While antenatal screening may benefit from further refinement and risk stratification, intrapartum ultrasound offers a promising pathway to enhance labour management, reduce reliance on invasive examinations, and improve maternal experience and clinical outcomes.

Automated measurements were feasible in all cases and acceptable in 90.4% of the initial assessments, reaching 100% after repeats were conducted. The 3D automated technique demonstrated good intra- and interobserver reproducibility and showed very good agreement with the manual technique. However, the 3D automated measurements were significantly wider than the manual measurements (133 ± 17° vs. 118 ± 21°, *p* = 0.013). While the assessment of the angle of progression (AoP) through 3D ultrasound was highly reproducible, the automated software systematically overestimated AoP compared to the manual technique, limiting its current clinical applicability [34]. The study found a strong correlation between the automatic and manual methods for measuring delta-HPD (ICC = 0.97) and delta-AoP (ICC = 0.99). The automatic algorithm demonstrated high accuracy, with a coefficient of determination (r^2^) of 0.98 for both measurements and low residual errors (1.2 mm for delta-HPD and 1.5° for delta-AoP). Bland–Altman analysis showed mean differences of 0.52 mm for delta-HPD and 0.35° for delta-AoP between the two methods, indicating good agreement [13]. The evidence suggests that ultrasound-based digital examination technologies offer potential for improving the accuracy of labour monitoring while enhancing patient comfort and potentially reducing complications.

The non-invasive purple line observation is used for vaginal examinations in various settings, and some invasive examinations like simple instruments for measuring cervical dilatation during labour with disposable instruments and Dila Check (interexaminer agreement) are also used [51,52]. The purple line method is a visual observation of the purple line as a proxy for cervical dilation and foetal head station. Finally, there are some studies on the non-invasive purple line observation, which has the potential to supplement the evaluation of labour progression [49,50,51,52,53,54,55,61]. Papoutsis et al.’s systematic review and meta-analysis found that the purple line appeared in 77.3% of women in active labour, with an occurrence ranging from 48.3% to 89.5%. There was a moderate positive correlation between the purple line’s length and cervical dilation (r = +0.64) and foetal head descent (r = +0.50). The mean length of the purple line was over 9.4 cm at 9–10 cm cervical dilation and over 7.3 cm at 3–4 cm dilation [67].

These findings suggest that the purple line may serve as a viable, non-invasive adjunct to vaginal examination (VE) for assessing labour progress. Its visibility, particularly during 1 cm dilation (47%), indicates its potential for clinical use, especially when minimising discomfort is a priority. However, its reliability across diverse populations and labour stages requires further validation. Similarly, the low-intensity light imaging probe represents an innovative approach to cervical dilation assessment. While early prototypes show promise, especially in low-light imaging, they currently lack robust clinical validation. Future research should focus on enhancing resolution, miniaturisation, and biocompatibility, and integrating machine learning for automated cervical assessment [55]. This scoping review highlights a growing interest in objective and less invasive labour monitoring technologies, with ultrasound and digital tools playing a central role. The emergence of novel devices reflects a shift away from traditional, subjective methods like VE toward more standardised and patient-friendly approaches. However, implementation challenges persist. These include the need for training and for the standardisation of ultrasound techniques, increased examination time and resource demands, and variable access to equipment, particularly in low-resource settings. Moreover, while technologies like the sonopartogram and transperineal ultrasound show high accuracy and patient preference, their integration into routine practice requires careful consideration of feasibility, cost, and long-term outcomes.

In conclusion, while innovative tools such as the purple line and light imaging probes offer exciting possibilities, ultrasound remains the most validated and widely accepted non-intrusive method for intrapartum monitoring. Continued research and investment are essential to refine these technologies and ensure their equitable, effective implementation across diverse clinical settings.

## 6. Strengths and Limitations of the Study

### 6.1. Strengths

This scoping review employed a comprehensive and systematic search strategy across multiple databases, ensuring broad coverage of the existing literature on both intrusive and non-intrusive ultrasound technologies used during intrapartum care. It adhered to a well-established methodological framework, enhancing transparency and reproducibility. The inclusion of diverse technologies ranging from manual ultrasound techniques to automated tracking algorithms allowed for a nuanced understanding of their accuracy, feasibility, and clinical relevance. The review also contextualised the findings within maternal care settings, highlighting patient comfort, diagnostic precision, and potential for reducing invasive procedures.

### 6.2. Limitations

Despite its strengths, the review was limited to studies published in selected languages, potentially excluding relevant findings from the non-English literature. Additionally, while the review mapped technological accuracy and feasibility, it did not fully explore implementation challenges such as infrastructure limitations, digital literacy among healthcare providers, and variability in access to ultrasound equipment, particularly in low-resource settings. The heterogeneity of the study designs and outcome measures also posed challenges in synthesising the findings. Furthermore, the clinical applicability of some advanced technologies remains constrained by the need for standardisation, training, and integration into routine workflows.

## 7. Conclusions and Recommendation

This scoping review highlights a significant shift toward ultrasound-based technologies, including transabdominal, transperineal, 2D, 3D, and automated approaches, as they are more accurate, reliable, and patient-friendly alternatives to traditional vaginal examinations (VEs). These methods consistently outperform VEs in assessing cervical dilation, foetal head station, and angle of progression, particularly during the second stage of labour. Emerging innovations such as purple line observation and low-intensity light imaging probes further reflect the growing interest in non-invasive, objective labour monitoring tools. However, many of these technologies remain in early development stages and require further validation.

In summary, the research landscape indicates a shift toward more objective and technology-driven approaches to labour management. While these technologies hold significant promise, careful planning, implementation, and ongoing evaluation are essential to ensure that they improve the quality of care and promote positive childbirth experiences for all women.

This synthesis of research highlights the potential of digital technologies to revolutionise labour monitoring while identifying gaps that require attention from practitioners, policymakers, and researchers alike.

This scoping review maps the accuracy of existing artefacts and devices developed to monitor labour progress. It finds that tools such as ultrasound and automated tracking algorithms demonstrate high accuracy and reproducibility, particularly for cervical dilation, foetal head station, and progression angle. Ultrasound surpasses vaginal examination (VE) in sensitivity, specificity, and patient comfort. While automated methods align well with manual techniques, limitations in clinical applicability and standardisation remain.

Critics note that none of the identified scoping studies reported accuracy in monitoring cervical effacement, moulding, and caput, nor did they address the latent and transition phases of labour monitoring. These limitations of the existing technologies indicate the need for further research for these methods to fully replace intrusive vaginal examinations.

## 8. Clinical Practice

The growing body of evidence supports the use of intrapartum ultrasound as a valuable adjunct to, and in some cases a potential replacement for, VEs. Ultrasound can provide more objective and accurate assessments of cervical dilatation, foetal head position, and foetal head descent. Combining ultrasound assessments with other clinical parameters may allow for more personalised labour management strategies tailored to the individual needs of each woman.

## 9. Policy Implications

Professional organisations and healthcare institutions should develop clear guidelines and training programmes for the appropriate use of intrapartum ultrasound and novel partogram designs. Healthcare policymakers need to consider the resources required to implement ultrasound-based labour management, including equipment costs, training of personnel, and infrastructure support. Efforts should be made to standardise ultrasound protocols to ensure consistency in labour assessment and management across different settings.

## 10. Research Implications

Future research should focus on conducting large-scale comparative effectiveness studies to evaluate the impacts of ultrasound-based labour management and novel partogram designs on maternal and neonatal outcomes. Cost-effectiveness analyses are needed to determine the economic value of implementing these technologies in different healthcare settings. Further research is needed to identify the barriers to and facilitators for the successful implementation of ultrasound-based labour management and novel partogram designs in clinical practice.

## Figures and Tables

**Table 2:** Ultrasound-based technologies used in monitoring the progress of labour and birth, characterised by study setting, sample size, study design, and technologies used for examination as compared to vaginal examination for the period of 2000 to 2025.

Study Title	Author and Year of Publication	Study Setting/Country	Sample Size	Study Design	Study Participants	Technology/Application/Devices Used
‘The sonopartogram: a novel method for recording progress of labour by ultrasound’	(Hassan et al., 2014) [5]	Two European maternity units (UK and Norwegian)	52	Prospective observational study	Women in labour	Sonopartogram
‘Implementation of sonopartogram: multicentre feasibility study’	(Lee et al., 2024) [31]	Hong Kong SAR, China	100	Prospective longitudinal cohort study	Women in labour	Integrating transabdominal and transperineal ultrasound
‘Intrapartum measurement of cervical dilatation using trans labial 3-dimensional ultrasonography: correlation with digital examination and interobserver and interobserver agreement assessment’.	(Zimerman et al., 2009) [48]	Israel	52	Prospective observational study	Women in labour	3D ultrasonographic
‘Transperineal ultrasonography for labour management: accuracy and reliability’	Yuce, Kalafat, & Koc, 2015) [47]	Türkiye	79	Prospective observational study	Women in labour	Transperineal ultrasonography
‘Automated Measurement of the Angle of Progression in Labor: A Feasibility and Reliability Study’	(Youssef et al., 2017) [46]	Italy	52	Non-consecutive series	Active labour	Angle of progression; transperineal, two-dimensional ultrasound; automated measurement
‘Agreement between two- and two-dimensional transperineal ultrasound methods for assessment of foetal head–symphysis distance in active labour’	Youssef et al., 2014) [45]	Italy	86	Non-consecutive series	Active labour	Two-dimensional (2D) and three-dimensional (3D) transperineal ultrasound methods
‘Sonographic parameters for diagnosing foetal head engagement during labour’	(Wiafe, Whitehead, Venables, & Odoi, 2018) [44]	Ghana	201	Prospective cross-sectional	Parturient	Ultrasound
‘Intrapartum ultrasound assessment of cervical dilatation and its value in detecting active labour’	(Wiafe, Whitehead, Venables, Dassah, & Eggebø, 2018) [43]	Ghana	195	Cross-sectional study	Women in labour	Ultrasound
‘Comparing intrapartum ultrasound and clinical examination in the assessment of foetal head position in African women’	(Wiafe, Whitehead, Venables, & Dassah, 2019) [42]	Ghana	196	Cross-sectional study	Women in active labour	Ultrasound
‘The feasibility and accuracy of ultrasound assessment in the labour room’	(Usman, Wilkinson, Barton, & Lees, 2019) [41]	UK	192	Prospective observational study	Women in labour	Transabdominal and transperineal ultrasound
‘Comparison between ultrasound parameters and clinical examination to assess foetal head station in labour’	(Tutschek, Torkildsen, & Eggebø, 2013) [40]	Norway	106	A prospective observational cohort study	Women in labour	Ultrasound
‘Agreement between two- and two-dimensional transperineal ultrasound methods in assessing foetal head descent in the first stage of labour’	(Torkildsen, Salvesen, & Eggebø, 2012) [39]	Norway	106	A prospective observational cohort study	Women in labour	Two-dimensional (2D) and three-dimensional (3D) transperineal ultrasound
‘Intrapartum foetal head position I: comparison between transvaginal digital examination and transabdominal ultrasound assessment during the active stage of labour’	(Sherer, Miodovnik, Bradley, & Langer, 2002) [38]	USA	102	Prospective study	Active labour	Transabdominal ultrasound
‘Sonographic assessment of the cervix before, during and after a uterine contraction is effective in predicting the course of labour’	(Saito et al., 2003) [37]	Japan	73	Cross-sectional study	Women who were admitted in labour	Transvaginal sonography
‘The diagnosis of foetal head engagement: Transperineal ultrasound, a new useful tool’	Rivaux et al., 2012) [36]	France	100	Prospective unicentric study	Women who were admitted in labour	Transperineal ultrasound
‘Correlation between clinical foetal head station and sonographic angle of progression during the second stage of labour’	(Perlman et al., 2018) [35]	Israel	70	Prospective observational study	Women in labour	Sonographic angle of progression
‘Automated 3D ultrasound measurement of the angle of progression in labour’	(Montaguti, Rizzo, Pilu, & Youssef, 2018) [34]	Italy	52	Non-consecutive series	Active labour	Automated 3D ultrasound
‘Assessment of labour progression by intrapartum ultrasonography among term nulliparous women’	(Mohan et al., 2019) [33]	India	215	Observational study	Women in labour	Ultrasonography
‘New technique for automatic sonographic measurement of change in head–perineum distance and angle of progression during active phase of second stage of labour’	Angeli et al., 2020) [13]	Italy	27	Prospective observational cohort study	Women in labour	Automatic sonographic measurement of changes in head–perineum distance and angle of progression
‘A new method to assess foetal head descent in labour with transperineal ultrasound’	(Barbera, Pombar, Perugino, Lezotte, & Hobbins, 2009) [14]	Italy	88	Prospective observational study	Labouring patients	Transperineal ultrasound
‘Automatic measurement of head-perineum distance during intrapartum ultrasound: description of the technique and preliminary results’	(Angeli et al., 2022) [12]	Italy	30	Non-consecutive series	Labouring women	Automatically measured by an innovative algorithm and manually by trained sonographers
‘Agreement between transperineal ultrasound measurements and digital examinations of cervical dilatation during labour’	(Benediktsdottir, Eggebø, & Salvesen, 2015) [15]	Sweden	86	Prospective observational study	Labouring women	2D transperineal ultrasound vs. vaginal examination
‘Automatic Evaluation of Progression Angle and Foetal Head Station through Intrapartum Echo graphic Monitoring’	Casciaro et al., 2013) [16]	Italy	10	A prospective observational cohort study design	Labouring women	Intrapartum echographic
‘Reliability and Reproducibility of Analyzing 3D Transperineal Ultrasound Volumes Obtained in the First Phase of Labor-A Pilot Study’	Caspers et al., 2023) [57]	Germany	54	A pilot study	Labouring women	3D transperineal ultrasound
‘Measurement of Angle of Descent (AOD) by Transperineal Ultrasound in Labour to Predict Successful Vaginal Delivery’	(Malik & Singh, 2020) [32]	India	64	Prospective observational study	Labouring women	Transperineal ultrasound
‘Sonographic measurement of cervical length and head perineum distance before labour to predict time of delivery’	(Chan et al., 2022) [17]	China	129	Observational study	Pregnancy +37wks completed	Transperineal sonographical
‘Use of a pocket-device point-of-care ultrasound to assess cervical dilation in labour: correlation and patient experience’	(Connell, Turrentine, & Antoniewicz, 2023) [18]	USA	30	Observational study	Labouring pregnant	Pocket-sized ultrasound
‘Automatic ultrasound technique to measure angle of progression during labour’	(Conversano et al., 2017) [19]	Italy	39	Prospective observational study design	Labouring pregnant	Translabial sonographic examination
‘Accurate evaluation of the progress of delivery with transperineal ultrasound may improve vaginal delivery: a single-center retrospective study’	(Enomoto et al., 2023) [20]	Japan	200	Retrospective study	Labouring pregnant	Transperineal ultrasound
‘Transperineal versus transvaginal ultrasound cervical length measurement and preterm labour’	(Gauthier et al., 2014) [56]	France	62	Prospective randomised study	Pregnant and labour	Transperineal versus transvaginal ultrasound
‘Intrapartum transperineal ultrasound assessment of foetal head progression in active second stage of labour and mode of delivery’	(Ghi et al., 2013) [21]	Italy	71	Prospective non-consecutive series	Labouring pregnant	Intrapartum transperineal ultrasound
‘Evaluation of sonographic assessment of the progress of labour’	(Głuszak, Dziadecki, Wielgoś, & Węgrzyn, 2015) [22]	Poland	83	Prospective observational cohort study	Labouring pregnant	Intrapartum sonographic
‘Transvaginal ultrasound of cervical length and its correlation to digital cervical examination, time to spontaneous labour and mode of delivery’	(Grotegut et al., 2011) [23]	Israel	726	Prospective cohort study	Labouring pregnant	Transvaginal ultrasound (TVUS)
‘A Novel Partogram for Stages 1 and 2 of Labor Based on Foetal Head Station Measured by Ultrasound: A Prospective Multicenter Cohort Study’	Haberman et al., 2021) [24]	France	613	Prospective multicenter cohort study	Labouring pregnant	Ultrasound
‘Intrapartum ultrasound at the initiation of the active second stage of labour predicts spontaneous vaginal delivery’	(Hadad et al., 2021) [25]	Israel	197	Prospective observational study	Labouring pregnant	Ultrasound
‘Intrapartum ultrasound for cervical dilatation: Inter- and intra- observer agreement’	(Hanidu et al., 2024) [26]	UK	206	Prospective longitudinal observational cohort study	Labouring pregnant	Transvaginal ultrasound (TVUS)
‘Simple two-dimensional ultrasound technique to assess intrapartum cervical dilatation: a pilot study’	Hassan, Eggebø, Ferguson, & Lees, 2013) [27]	Belgium	21	Prospective observational multicenter study	Labouring pregnant	Two-dimensional (2D) ultrasound
‘Quantitative sonoelastography of the uterine cervix prior to induction of labour as a predictor of cervical dilation time’	(Hee, Rasmussen, Schlütter, Sandager, & Uldbjerg, 2014) [28]	Denmark	73	Cross-sectional study	Labouring pregnant	Quantitative sonoelastography
‘Reliability of transperineal ultrasound for the assessment of the angle of progression in labour using parasagittal approach versus midsagittal approach’	(Kamel et al., 2021) [29]	Italy	151	Non-consecutive series	Women in active labour	Transperineal ultrasound
‘A longitudinal study investigating cervical changes during labour using a wireless ultrasound device’	Kim, Jeon, & Jung, 2018) [30]	South Korea	25	Longitudinal study	Women in active labour	Wireless mobile US device (SONON)

**Table 3:** Key findings of non-invasive methods (ultrasound-based technologies) compared to invasive methods (vaginal examination) for monitoring the progress of labour and labour follow-up.

Author and Year	Aims and Objectives	Conclusion and Recommendations	Strengths and Limitations
(Hassan et al., 2014) [5]	The study assesses labour progress using digital vaginal examination (VE), and ultrasound (US) a non-intrusive ultrasound-based assessment (sonopartogram) to evaluate cervical dilatation and foetal head descent and rotation.	– The study found that the sonopartogram was more effective at assessing labour progress than the DVE. – The digital VE and US agreement was good for cervical dilatation and foetal head rotation, but less so for head descent.	
(Lee et al., 2024) [31]	‘To evaluate the feasibility of systematically integrating transabdominal and transperineal ultrasound assessment of foetal position, parasagittal angle of progression (psAOP), head–perineum distance (HPD) and sonographic cervical dilatation (SCD) to monitor the progress of labour in women undergoing induction of labour (IOL) and to determine if ultrasound can alleviate pain’.	– Ultrasound offers a reliable, reproducible method for monitoring labour progress. This study pioneers the full integration of ultrasound to replace some vaginal examinations, using combined transabdominal and transperineal approaches. The technique is feasible, reduces maternal discomfort, and supports a shift toward objective, safer, and more patient-friendly intrapartum care.	This study has strengths such as a longitudinal design, multiple transabdominal and transperineal ultrasound measurements, and low intersonographer variability. However, it has limitations, such as a small sample size and findings that may not be generalizable.
(Zimerman et al., 2009) [48]	‘To determine the accuracy and reproducibility of intrapartum translabial 3-dimensional (3D) ultrasonographic measurements of cervical dilatation during labour’.	– Translabial 3D ultrasonography is a feasible and reproducible method for assessing cervical dilatation during labour. While further research is needed for routine clinical use, it offers a promising alternative to digital vaginal examinations, especially in low-risk cases and in the conservative management of premature labour, and supports pelvic examination training.	The study introduces a new intrapartum ultrasonographic assessment for cervical dilatation, providing a more comprehensive labour profile. This preliminary study, with a small number of cases, suggests further research is needed in specific clinical settings and different study designs.
Yuce, Kalafat, & Koc, 2015) [47]	‘To compare ultrasound measurements and clinical assessments of cervical dilatation, foetal head station and foetal head position’	– Ultrasound assessment during labour shows good agreement with clinical evaluation of cervical dilatation and moderate agreement with foetal station, but less so for head position. It reduces inter-rater variability and clinical examination needs, offering a reliable, accessible, and easy-to-learn alternative for monitoring labour progression, especially in early- and mid-labour stages.	Transperineal ultrasonography, a non-invasive method, is believed to be unaffected by complications due to its ability to produce images of cervical dilatation, head position, and head station.
(Youssef et al., 2017) [46]	‘To assess the feasibility and reliability of a new automated method for the measurement of the angle of progression (AoP) in labour’.	– Automated measurements were feasible and reproducible, showing good intra- and interobserver reproducibility. – However, the automated software’s measurements differed significantly from those from the manual technique. – Based on the data, the automated technique is not yet ready for clinical use, and the AoP should be exclusively measured using the manual technique.	
Youssef et al., 2014) [45]	‘To assess the intermethod agreement between two-dimensional (2D) and three-dimensional (3D) transperineal ultrasound methods in measuring a new index of foetal head station (the foetal head–symphysis distance (HSD) in active labour, and to assess potential factors that may affect their agreement’.	– The study shows excellent agreement between 2D and 3D methods for measuring head–symphysis distance (HSD), a simple measurement between the maternal symphysis pubis and the foetal skull. – It has high intra- and interobserver reproducibility, even on slightly parasagittal measurements. – Foetal HSD measurements have excellent intermethod agreement between 2D and 3D techniques, with the 2D technique being simpler and more widely available, making it preferred in labour rooms.	The study did not consider the integrity of foetal membranes, which could influence head–symphysis distance measurements.
(Wiafe, Whitehead, Venables, & Odoi, 2018) [44]	‘To investigate the diagnostic performance of the head–perineum distance, angle of progression, and the head–symphysis distance as intrapartum ultrasound parameters in the determination of an engaged foetal head’	– This study investigates the use of the head–symphysis distance (HSD) as an additional sonographic parameter to complement the angle of progression (AoP) and head–perineum distance (HPD) in determining engaged foetal heads. – The results suggest that high agreement among these parameters may increase confidence in using ultrasound for foetal head engagement. – This is the first report on ultrasound diagnostic performance in detecting engaged foetal heads in a black African population.	Thus, this article also adds important knowledge about using ultrasound in labour in other populations.
(Wiafe, Whitehead, Venables, Dassah, & Eggebø, 2018) [43]	‘To examine the agreement between ultrasound and digital vaginal examination in assessing cervical dilatation in an African population and to assess the value of ultrasound in detecting active labour’.	– Ultrasound effectively assesses cervical dilatation and may help detect active labour. However, study design limitations and varying labour ward admission practices, especially in Africa, affect its applicability. Further research is needed on precision, infection risks from VE, and maternal preferences to optimise ultrasound use in labour monitoring.	VE may be limited to cases that cannot be visualised by ultrasound, reducing maternal discomfort and infection risks. Additionally, most women are in active labour when examined, which may reduce the external validity of the study, and late arrivals in high-resource countries may also cause this.
(Wiafe, Whitehead, Venables, & Dassah, 2019) [42]	‘To examine the agreement between intrapartum ultrasound and digital vaginal examination in assessing the occiput position in black African women who were in the first stage of labour and to evaluate the influence of ruptured membranes on this agreement’	– The study reveals that ultrasound and VE have poor agreement on occiput posterior position in black African women during the first stage of labour. – Over 85% of foetal head positions cannot be determined by VE, indicating that VE has difficulty in detecting malposition and that ultrasound is more useful for such cases. – The study concluded that ultrasound is more useful than VE for detecting malposition in the first stage of labour, with over 85% of foetal head positions not determined by VE being occiput transverse and posterior positions.	
(Usman, Wilkinson, Barton, & Lees, 2019) [41]	‘To assess the feasibility and accuracy of transabdominal and transperineal ultrasound compared to vaginal examination in the assessment of labour and its progress’.	– The study compares the feasibility and accuracy of transabdominal and transperineal ultrasound (US) and vaginal examination (VE) in labour assessment. Results show that neither technique has superiority. – VE is poor at determining foetal head position, with its low accuracy not improving with increasing cervical dilatation. – Ultrasound is useful in early labour but less effective in active labour. – VE is subjective and significantly affected by caput, making it less effective in head station assessment.	This study reports the largest number of assessments of intrapartum ultrasounds to date. The large, ethnically diverse cohort improves generalizability. Many assessments were excluded due to the US scan and VE being performed over 30 min apart.
(Tutschek, Torkildsen, & Eggebø, 2013) [40]	‘To analyse the relationship between ultrasound parameters such as intrapartum transperineal ultrasound (ITU) head station, angle of progression, head-perineum distance, and head-symphysis distance, and compare them with DVE during labour’.	– The study suggests that infrapubic ultrasound can be used to assess birth progress using sonographic markers such as intrapartum transperineal ultrasound (ITU) foetal head station, angle of progression (AOP), foetal head–perineum distance (HPD), and foetal head station (HSD). – These parameters are more reproducible than digital vaginal examination and should be considered if there are uncertainties about birth progress or if DVE is impaired. – Feedback from labouring women suggests that ultrasound methods may be perceived as less uncomfortable than digital examination. – The ultrasound parameters showed a high degree of correlation with each other, but only moderate correlation with vaginally examined foetal head station.	The ultrasound parameters showed a high degree of correlation with each other, but only moderate correlation with vaginally palpated foetal head station.
(Torkildsen, Salvesen, & Eggebø, 2012) [39]	‘To study intraobserver repeatability and intermethod agreement between two- (2D) and two dimensional (3D) transperineal ultrasound methods in assessing foetal head descent during the first stage of labour’.	– The study found that for one ultrasound operator, the intraobserver repeatability and agreement between 2D and 3D ultrasound methods in the prolonged first stage of labour were good. – Two-dimensional methods were simpler to learn and could be quickly analysed online, making them a preferred choice for labour room use. – This suggests that 2D equipment may be more effective in the labour room.	
(Sherer, Miodovnik, Bradley, & Langer, 2002) [38]	‘To determine the correlation between transvaginal digital and the gold standard technique of transabdominal suprapubic ultrasound assessments of foetal head position during labour’.	– In summary, this study demonstrates an overall high rate of error in foetal head position determinations by transvaginal digital examination. – These data support the idea that intrapartum application of transabdominal ultrasound may significantly enhance correct determination of foetal head position during active labour. – Intrapartum ultrasound may therefore potentially be utilised as an educational tool to assist physicians in training.	The study’s cross-sectional design is a potential weakness.
(Saito et al., 2003) [37]	‘To investigate whether the degree of change in cervical length during a uterine contraction is predictive of subsequent progression of labour’.	– Sonographic cervical assessment during and between contractions effectively predicts labour progression. Real-time ultrasound helps distinguish inefficient from normal contractions, aiding timely management. Cervical shortening, especially during normal contractions, supports its use. Measuring cervical length dynamically offers a reproducible, accessible tool to guide labour decisions and improve outcomes.	
Rivaux et al., 2012) [36]	‘Assessment of foetal head engagement by digital examination is highly subjective even though this method remains the gold standard. Ultrasonography could be helpful to determine foetal head engagement during the second stage of labour’.	– Abdominal intrapartum ultrasound increases the accuracy of foetal head position assessment. – Translabial ultrasound is a simple and easy method to define foetal head engagement by measuring the distance between the perineum and foetal head. – Ultrasound during the second stage of labour may serve as an educational tool for physicians in training.	
(Perlman et al., 2018) [35]	‘To investigate the correlation between the angle of progression and the clinical foetal head station (FHS) during the second stage of labour, and to build reference range’.	– The study found a significant correlation between clinical foetal head station (FHS) and the transperineal ultrasound (TPU)-measured angle of progression (AOP), suggesting that these standardised sonographic values can be used as an objective tool for FHS evaluation during labour. – These standardized sonographic values can serve as a reliable, objective tool for evaluating the FHS during the second stage of labour.	
(Montaguti, Rizzo, Pilu, & Youssef, 2018) [34]	‘To assess the feasibility and reliability of an automated technique for the assessment of the angle of progression (AoP) in labour using two-dimensional (3D) ultrasound’	– The study demonstrates that automated software is highly reproducible for measuring the angle of progression (AoP) on 3D ultrasound acquisitions. – However, it leads to systematic overestimation of AoP compared to the standard manual technique, which is hindering its use in clinical practice. – This overestimation can result in false diagnoses of low foetal head stations, potentially leading to complications like failed instrumental delivery or foetal and maternal injuries. – The study concludes that automated software is not yet adequate for assessing AoP in clinical practice.	
(Mohan et al., 2019) [33]	‘To assess cervical dilation, foetal head station, and foetal head position by intrapartum ultrasonography and to compare the approach with digital vaginal examination (DVE)’.	– In conclusion, intrapartum ultrasound was found to be a useful tool for monitoring labour progression during the active phase among term nulliparous women. – The agreement between DVE and ultrasound findings was strong for cervical dilation, and fair for foetal head station and head position. – Most labouring women reported that ultrasonography was less distressing and preferred it over DVE for assessment of labour. – Intrapartum ultrasonography was preferred as an objective assessment tool for labour progression among term nulliparous women and therefore should be practiced in all labour rooms. – Further studies on interobserver variation are recommended to establish the reproducibility of intrapartum assessment by ultrasonography. – Intrapartum ultrasonography is recommended for labour progression assessment in term nulliparous women, and further studies on interobserver variation are recommended.	The study’s strengths include a small sample size, consistent observers, and comparing outcomes between VE and ultrasonography for labour progression.
Angeli et al., 2020) [13]	‘To evaluate the performance of a new ultrasound technique for the automatic assessment of the change in head–perineum distance (delta-HPD) and angle of progression (delta-AoP) during the active phase of the second stage of labour’.	– The automatic assessment of the delta-angle of progression (delta-AoP) and delta-head–perineum distance (delta-HPD) during maternal pushing efforts is feasible. – The automatic measurement of delta-AoP appears to be reliable when compared with the gold-standard manual measurement by an experienced operator.	Further studies are required to confirm the accuracy of this automatic tool and to demonstrate its clinical advantages in a routine setting
(Barbera, Pombar, Perugino, Lezotte, & Hobbins, 2009) [14]	‘To assess the feasibility and reproducibility of measuring foetal head station and descent during labour using transperineal ultrasound (TPU) imaging, to compare the evaluation of foetal station through digital examinations with concurrent TPU assessments, and to assess its utility in distinguishing patients whose pregnancy will result in spontaneous vaginal delivery from those who will require operative vaginal delivery or Caesarean section for failure to progress’	– In conclusion, the use of transperineal ultrasound (TPU) examination to measure the angle of head descent is an objective, reproducible, non-invasive, and easily performed technique that uses precise landmarks to assess true foetal head station. – We are hoping that, after further investigation, transperineal ultrasound (TPU) imaging may have the potential to be used as a more accurate tool in decision-making in the event of true failure to progress in labour and that it may aid in guiding clinicians with regard to operative vaginal delivery.	
(Angeli et al., 2022) [12]	‘To evaluate the accuracy and reliability of a new ultrasound technique for the automatic assessment of the head-perineum distance (HPD) during childbirth’.	– The study demonstrates the feasibility of automated measurement of head–perineum distance during intrapartum ultrasound, showing good correlation with gold-standard manual measurements by senior experts. – This new algorithm has the potential to support clinicians in performing intrapartum ultrasounds, which have been suggested to improve labour management. – The automatic algorithm for assessing head–perineum distance is a reliable technique.	
(Benediktsdottir, Eggebø, & Salvesen, 2015) [15]	‘To compare 2D transperineal ultrasound assessment of cervical dilatation with vaginal examination and to investigate intra-observer variability of the ultrasound method’.	– The study found that transperineal ultrasound is a suitable method for assessing cervical dilatation during the first stage of labour. – However, it was found that in 65% of women, cervical dilatation could not be measured when the cervix was ≥8cm dilated. – The ultrasound method had an average 1 cm less intraobserver repeatability than digital assessment. – The study concluded that transperineal ultrasound is a suitable method for assessing cervical dilatation in latent and early active labour stages, and that replacing some clinical examinations with transperineal ultrasound examinations might decrease infection risk.	Longitudinal studies are needed.
Casciaro et al., 2013) [16]	‘Aim of this work is the preliminary clinical validation and accuracy evaluation of our automatic algorithm in assessing progression angle (PA) and foetal head station (FHS)’	– This study validates a novel, minimally invasive ultrasound method using automatic tracking algorithms to monitor labour progression. Tested on a simulator and clinically validated, it accurately measures foetal head station and progression angle across all labour phases. The technique offers objective, quantitative data, enhancing childbirth monitoring while reducing maternal and foetal discomfort.	The automated algorithms evaluated could address the need for new standardised quantitative monitoring approaches
Caspers et al., 2023) [57]	‘To investigate the reliability and reproducibility of transperineal ultrasound (TPUS) in the initial phase of labour. The study aimed to evaluate the reliability and reproducibility of transperineal ultrasound (TPUS) during labour, a common method that can supplement or replace DVE and used a 4-dimensional method for cervical effacement assessment’.	– The study suggests that transperineal ultrasound (TPUS) can supplement or replace DVE in certain situations. – It used a four-dimensional method for cervical effacement assessment; the study found TPUS to be a valuable non-invasive tool with good diagnostic accuracy for angle of progression, cervical length, and dilatation. – While it should not replace VE, TPUS could serve as an alternative method for monitoring labour progression in the future. – This study provides support for the use of TPUS to complement a vaginal examination.	
(Malik & Singh, 2020) [32]	‘To measure the Angle of Descent (AOD) by transperineal ultrasound in labour to predict the possibility of a successful vaginal delivery’.	– The study uses transperineal ultrasound to measure the angle of descent (AOD) in labour to predict the possibility of a successful vaginal delivery. – It found that an increased AOD decreased the time interval to delivery. – The study suggests that using intrapartum transperineal ultrasound and measuring AOD can be beneficial in managing labour, especially in prolonged stages. – The authors recommend using ultrasound in labour rooms due to its non-invasive nature and informational values. – TPUS is a valuable non-invasive tool with good diagnostic accuracy for the AoP, cervical length, and dilatation.	
(Chan et al., 2022) [17]	‘This was to measure cervical length and head perineum distance and the prediction of time of delivery’	– In conclusion, transperineal sonographical assessment of cervical length and head–perineum distance before labour was not useful in predicting the time of delivery. – However, it can be explored as an alternative assessment method when a digital vaginal examination is not preferred. – The study found that transperineal sonography was not effective in predicting delivery time before labour onset, as labour is a gradual process influenced by the release of prostaglandin and its effects on cervical tissue and uterine muscles.	
(Connell, Turrentine, & Antoniewicz, 2023) [18]	‘To estimate the correlation of cervical dilation between pocket-device point-of-care transperineal ultrasound (TPUS) and digital cervical examination (DCE)’.	– Measurement of cervical dilation using a pocket-device point-of-care TPUS has a strong positive correlation with DCE and offers a non-invasive, convenient alternative to traditional digital exams in term, labouring patients. – Its function is focused on identifying acute diagnostic outcomes, not conducting a comprehensive examination. – Future research and policy development are needed to optimise care during the intrapartum period and ensure the use of this new technology.	The device is portable but easily lost if not secured. Although portable, there are no current national regulatory guidelines for this ultrasound device’s modality in obstetrics and gynaecology, particularly for cervical dilation assessment.
(Conversano et al., 2017) [19]	‘To evaluate the accuracy and reliability of an automatic ultrasound technique for assessment of the angle of progression (AoP) during labour’.	– In conclusion, the translabial ultrasound approach presented in this paper is well-tolerated by patients and the automatic algorithm makes it less operator-dependent, allowing objective quantification of the AoP with a high level of accuracy. – This automatic technique has the potential to reduce human error and speed up ultrasound acquisition time, which should facilitate monitoring of the progress of labour and ultimately decision-making regarding delivery options. – Future studies will focus on extended clinical validation combined with the simultaneous measurement of other labour monitoring parameters through the same approach.	The proposed automatic algorithm is a reliable technique for measurement of the angle of progression (AoP). Its (relative) operator-independence has the potential to reduce human errors and speed up ultrasound acquisition time, which should facilitate management of women during labour.
(Enomoto et al., 2023) [20]	‘To clarify the impact of introducing Transperineal ultrasound (TPU) on perinatal outcomes at Mie University Hospital’.	– The study suggests that transperineal ultrasound (TPU) can increase the rate of vaginal delivery by accurately evaluating the descent of the foetal head station. – This can prevent underestimation of delivery progress and reduce emergency caesarean sections. – Transperineal ultrasound (TPU) evaluation can also serve as a basis for retrospective reviews and provide valuable feedback for clinical settings. – The rate of vaginal deliveries increased significantly from 2019 to 2020, suggesting the introduction of TPU may have contributed to overall improvements in digital evaluation techniques.	This retrospective study had limitations, including there being no significant difference in labour arrest percentages or anaesthesia use rates. A prospective study should be conducted to further investigate these findings.
(Gauthier et al., 2014) [56]	‘To evaluate the agreement between and the reproducibility of transperineal and transvaginal ultrasound cervical length measurements performed by the duty obstetrical team in case of preterm labour’	– The study compared transperineal and transvaginal ultrasound for cervical length measurement and preterm labour. – It concluded that transperineal sonography is valid for preterm labour and acceptable for cervical assessment when a vaginal examination is contraindicated, such as in cases of placenta praevia, active bleeding, or poor patient tolerance.	In case of preterm labour, cervical length measurement with transperineal ultrasonography seems reproducible and can be performed by the obstetric team on duty.
(Ghi et al., 2013) [21]	‘To compare longitudinal changes in angle of progression (AoP) and midline angle (MLA) during the active second stage of labour according to the mode of delivery’	– This study offers original data on foetal head progression in the second stage of labour using 3D ultrasound, demonstrating that poor foetal head descent or delayed foetal head rotation can be accurately documented using serial intrapartum transperineal ultrasound. – Ultrasonographic assessment of foetal head descent in the second stage of labour may play a role in the prediction of the mode of delivery.	This study offers original data on foetal head progression in the second stage of labour using 3D ultrasound, but there are some limitations due to there being a limited number of patients who underwent operative delivery, preventing separate analysis of data from vacuum extraction and caesarean delivery.
(Głuszak, Dziadecki, Wielgoś, & Węgrzyn, 2015) [22]	‘To evaluate the practical application of intrapartum sonographic assessment of the progress of labour’.	– Ultrasonography may be useful in assessing the progress of labour as well as in predicting or early diagnosis of abnormal foetal head descent. – The inter-group difference in foetal head–perineum distance was noticeable but non-significant. – The study also found a relationship between these measured values and the time to second labour phase completion.	
(Grotegut et al., 2011) [23]	‘To determine if transvaginal ultrasound (TVUS) examination of cervical length correlates to digital pelvic examination and if it can predict time to and mode of delivery in term pregnancies’	– In summary, this study demonstrates that transvaginal ultrasound (TVUS) of the cervical length at term poorly correlates with digital cervical dilatation and effacement, but transvaginal ultrasound (TVUS) can predict the mode of delivery, with women eventually delivering via caesarean having a longer TVUS cervical length measurement at term. – In contrast, digital cervical dilatation is able to predict time to spontaneous delivery at term, with women with a more dilated cervix delivering sooner. – The study found a significant relationship between TVUS and effacement, but poor goodness of fit. Women with vaginal delivery had shorter cervixes determined by TVUS at 40 weeks compared to those delivering by caesarean section. – Digital cervical dilatation was found to predict time to spontaneous delivery.	The study reveals that transvaginal ultrasound can predict time to and mode of delivery in term pregnancies, but its role in predicting delivery mode and cervical length is not well established.
Haberman et al., 2021) [24]	‘To describe continuous labour curves, including second stage, based on foetal head station’	– The study presents a new partogram for labour stages 1 and 2, based on ultrasound measurements of the foetal head station. – The study emphasises the need for better understanding of the second stage, beyond static measurements like duration and waiting time. – It suggests that in patients with an unengaged foetal head, labour can continue for up to 3.8 h in nulliparous women and 3 h in multiparous women. – The study also suggests further research is needed to evaluate the value of non-invasive station-based labour curves on labour management and outcome.	
(Hadad et al., 2021) [25]	‘To evaluate whether foetal head station and position, as assessed by ultrasound at the beginning of the pushing process, can predict the mode of delivery and duration of pushing in nulliparous women’	– Ultrasound performed at the beginning of the active second stage of labour can assist in predicting the mode of delivery and duration of pushing, and performs better than the traditional digital exam, with angle of progression at rest and the delta-angle of progression being the best predictors. – Ultrasound parameters like the angle of progression and head–perineum distance are commonly used to assess foetal head station during the second stage of labour. – The head–symphysis distance is a good predictor of spontaneous vaginal delivery (SVD) in women with a prolonged second stage. – Studies have found a correlation between these parameters and labour duration, but the study found no significant difference between spontaneous and operative deliveries. – The AOP was found to be a better predictive tool compared to head–perineum distance and head–symphysis distance, due to better interobserver variability and accuracy.	
(Hanidu et al., 2024) [26]	‘To investigate the relationship between cervical dilatation as assessed by TPUS and DVE. To assess inter- and intra- observer variability in both single and repeated ultrasound assessments of cervical dilatation during active labour’	– The study suggests that intrapartum transperineal ultrasound (TPUS) can be used to assess the cervix during active labour, with a strong correlation with vaginal examination (VE) measurements. – The results support the use of intrapartum transperineal ultrasound (TPUS) as a reliable method for evaluating the cervix at various dilatations, with high consistency between DVE values and TPUS measurements of cervical dilatation with relatively low inter- and intraobserver variability.	Transperineal ultrasound enables visualisation and objective measurement of the cervical rim during labour across varying cervical dilatations and irrespective of membrane status.
Hassan, Eggebø, Ferguson, & Lees, 2013) [27]	‘To describe a two-dimensional (2D) ultrasound technique to measure cervical dilatation in labour, and to compare ultrasound with DVE measurements’.	– A pilot study demonstrates the feasibility of a two-dimensional (2D) ultrasound technique for measuring cervical dilatation during labour, with a close agreement between the technique and VE. – This technique could be an essential component of ‘sonopartograms’ for ultrasound assessment of labour progress, providing a more accurate and reliable assessment of cervical dilatation.	
(Hee, Rasmussen, Schlütter, Sandager, & Uldbjerg, 2014) [28]	‘To evaluate how the approximate Young’s modulus of the uterine cervix assessed by quantitative sonoelastography in patients undergoing induction of labour is associated with the cervical dilation time and to evaluate the approximate Young’s modulus as a predictor of prolonged cervical dilation time’.	– A new method using quantitative sonoelastography of the uterine cervix to predict cervical dilation time is presented for women admitted for induction of labour. – This method can supplement Bishop’s score and cervical length assessment when planning induction methods. – Improvements in the reference cap and a mechanical device with standardised compression and decompression cycles may enhance the technique’s performance.	This method may supplement Bishop’s score and the cervical length measurements concerning the prediction of cervical dilation time and the risk of prolonged dilation time after induction of labour.
(Kamel et al., 2021) [29]	‘To assess the inter-method agreement between midsagittal (msAoP) and parasagittal (psAoP) measurements of the angle of progression (AoP) during labour. In addition, we aimed to evaluate the correlation between AoP measurements by both midsagittal and parasagittal approaches with the mode of delivery’.	– The study found a significant difference in the measured angle of progression (AoP) between the parasagittal (psAoP) and midsagittal (msAoP) during labour data. – The automated measurements of AoP are designed using parasagittal visualisation of the echogenic pubic arch, not the hypoechogenic pubic symphysis. – The researchers suggest caution when applying data from midsagittal measurements in centres using the parasagittal automated approach.	The limitation of our study is that we have not evaluated intraobserver and interobserver variability
(Kim, Jeon, & Jung, 2018) [30]	‘Cervical assessment during digital vaginal examination (DVE) includes assessing cervical dilatation, effacement, position and consistency. Only cervical dilatation during labour has been previously researched. We investigated cervical changes, including cervical dilatation and effacement, using a wireless ultrasound (US) device’.	– This longitudinal study used a wireless US device to study cervical changes during labour. – This study using a wireless ultrasound device found a strong correlation between intrapartum sonography and vaginal examination (VE) for assessing cervical changes during labour, including cervical dilatation and thickness. – However, the high failure rate of cervical length image collection made it difficult to determine the correlation between cervical length and effacement. – A new technique for evaluating effacement with cervical thickness was developed, but further research is needed to define its role in labour wards.	Cervical dilation and thickness were found to correlate with VE findings. The device’s convenience in the labour ward could be beneficial, but further research is needed.

**Table 4:** Other emerging technologies used in monitoring the progress of labour and birth follow-up.

Study Title	Author and Year of Publication	Study Setting/Country	Sample Size	Study Design	Study Participants	Technology/Application/Devices Used
‘Use of the purple line to diagnose cervical dilatation and foetal head station during labour’	(Nunes, Locatelli, & Traebert, 2018) [53]	Brazil	220	Prospective cohort study	Women in labour	Purple line
‘The purple line as a measure of labour progress: a longitudinal study’	Shepherd et al., 2010) [54]	Scotland	144	Longitudinal observational study	Women in labour	Purple line
‘Development of a Low-Intensity Light Imaging Probe for Childbirth Cervical Dilation Image Acquisition’	(Takpor, Atayero, Adetiba, & Badejo, 2023) [55]	Nigeria	2880	Design is a proof-of-concept laboratory investigation	Women in labour	Prototype of a novel low-intensity light imaging probe
‘Simple instrument for measuring cervical dilatation during labour’	(Letić, 2005) [51]	Serbia and Montenegro		Proof-of-concept laboratory-based investigation	Women in labour; pregnant	Design of disposable clips, a measuring tape, and a flexible tube
‘The Diagnostic Accuracy of Purple Line in Prediction of Labor Progress in Omolbanin Hospital, Iran’	Kordi, Irani, Tara, & Esmaily, 2014) [50]	Iran	350	Cross-sectional study	Women in labour; pregnant	Purple line
‘Relationship between length and width of the purple line and foetal head descent in active phase of labour’	(Irani, Kordi, & Esmaily, 2018) [49]	Iran	350	Longitudinal observational study	Women in labour; pregnant	Purple line
‘Novel device vs. manual examinations for the measurement of cervical dilation in labour: a randomized controlled trial’	(Martin, Firman, & Berghella, 2021) [52]	Australia	42	A randomised controlled trial	Women in labour; pregnant	Dila Check (interexaminer agreement)

**Table 5:** Key findings on other emerging technologies and novel prototypes used for monitoring the progress of labour and birth follow-up.

Author and Year	Aims and Objectives	Conclusion and Recommendations	Limitations
(Nunes, Locatelli, & Traebert, 2018) [53]	‘To estimate the incidence of the purple line and if it can supplement the evaluation of labour progression’.	– In conclusion, the purple line was recorded frequently in women during labour, especially in patients of white ethnicity and after premature rupture of membranes. – The occurrence of the purple line was associated with adequate evolution of labour and presented a positive correlation with the parameters of labour progression.	It was not suitable for routine diagnostic use owing to its low accuracy.
Shepherd et al., 2010) [54]	‘To assess in what percentage of women in labour a purple line was present, clear and measurable and to determine if any relationship existed between the length of the purple line and cervical dilatation and station of the foetal head’.	– The purple line, a tool used to measure labour progress, has been found to have a medium positive correlation with cervical dilatation and foetal head station. – This line could be a useful guide for clinicians, but further research is needed to determine its clinical usefulness and its acceptability for both women in labour and clinicians. – The study suggests that the purple line’s measurement should be acceptable to both parties, as any reliable measurement of labour progress must be acceptable to both parties.	
(Takpor, Atayero, Adetiba, & Badejo, 2023) [55]	‘To provide a more accurate and less subjective way to monitor cervical dilation during labour by using a visual imaging technique instead of manual finger examination’	– The research showcases the use of optical imaging in acquiring images of cervical dilation simulation models. – A prototype low-intensity light imaging probe was developed for image acquisition, and the pre-processed images were compared to bright-light images. – The results showed that the pre-processed low-light images were similar to the standard cervical dilation models, demonstrating the potential of optical imaging in improving cervical dilation images. – This research demonstrated the use of a low-intensity light imaging probe as a possible objective alternative to the subjective insertion of fingers in vaginal examination. – A low-intensity light imaging probe for childbirth cervical dilation image acquisition has been developed. – The probe, which has a visualisation percentage of 47%, will be used for further development. – The probe will be miniaturised to a diameter of 3mm for easy insertion and will be equipped with a higher resolution camera with a low-light intensity of 50 lux. An improved biocompatible prototype will be developed for clinical studies.	Further research on machine learning and deep learning classification tasks will be explored for automated cervical dilation assessment.
(Letić, 2005) [51]	‘To develop simple instrument for measuring cervical dilatation during labour’	– A simple instrument for measuring cervical dilatation during labour is described, consisting of two clips, a measuring tape, and a flexible tube. – The instrument measures the differential displacement of markings on the tape and tube. Laboratory measurements were conducted to determine the error of measurement, with an absolute error of 1.5 mm. – Clinical measurements were performed on 12 parturients with a single foetus in vertex position and 3 to 4 cm cervical dilatation. – Results were within a ±1cm error margin of manual assessments, with minor discomfort to patients during application. – The study analysed the accuracy of a new instrument for measuring cervical dilatation. Out of 12 parturients, it was successfully applied to 10, with 2 unsuccessful applications occurring during the first application. – The instrument’s application time was shorter for subsequent applications, and it was found that pelvic examinations performed as if the instrument was not present caused the instrument to become entangled. – Testing the instrument’s accuracy in measuring enlargement of cervical dilatation was not possible due to the displacement of the measuring tape by the presenting part of the foetus. – The instrument’s simplicity, cost-effectiveness, and ease of use make it a suitable choice for disposable use.	
Kordi, Irani, Tara, & Esmaily, 2014) [50]	‘To determine the diagnostic accuracy of purple line in the prediction of labour progress’	– The purple line, found in 75.3% of women during labour, has high sensitivity and specificity in predicting labour progress. It has a 90.2% sensitivity in the first stage and 87.6% in the second, and 68.57% in total labour. – This makes it a useful non-invasive method for clinical assessment to check on the progress of labour. – The study examines the diagnostic accuracy of the purple line in predicting labour progress and acknowledges the limitations of subjective measurements like cervical dilatation and foetus head descent, and it is the gold standard of vaginal examination. – The study suggests that the purple line’s high sensitivity and specificity make it a suitable non-invasive method for labour progress assessment.	
(Irani, Kordi, & Esmaily, 2018) [49]	‘To assess the relationship between length and width of the purple line and foetal head descent’	The study shows a positive correlation between the purple line and foetal head station, suggesting it as a non-invasive alternative to vaginal examinations, which are often painful and intrusive. This method may reduce unnecessary exams and improve labour monitoring. Further research is needed to confirm its reliability across different ethnic groups.	This study has limitations due to subjective labour progress assessments and lack of accurate equipment, even though only one midwife performs the entire assessment to minimise confounding factors.
(Martin, Firman, & Berghella, 2021) [52]	‘To evaluate the agreement among different providers’ examinations using DilaCheck (interexaminer agreement) compared with interexaminer agreement between 2 manual examinations for cervical dilation of women in labour’.	A randomised trial evaluating the Dila Check device found it did not improve interobserver agreement in cervical dilation measurements. Manual exams showed better agreement within 1 cm but remained imprecise overall. The study highlights the need for continued innovation in labour monitoring tools to reduce discomfort and improve accuracy in clinical decision-making.	

## Data Availability

All data generated or analysed during this study are included in this article and its Supplementary Materials.

## Supplementary Materials

The following supporting information can be downloaded at: https://www.mdpi.com/article/10.3390/healthcare14152359/s1; Search supplementary file1. Data extraction instrument.xlsx file 2; supplementary (PRISMA-ScR) Checklist file 3 [68]; Supplementary data set .xlsx file 4.

## Appendix A. Search Strategy

The search strategy used for the PubMed database with the Boolean terms OR/AND and truncation is as follows: (labour women OR delivery women OR childbirth women OR intrapartum women) AND (non-invasive assess OR devices for Measuring) OR examinations) AND digital vaginal) OR digital) AND vaginal) OR vaginal examination) OR Artificial Assisted intelligence) OR AI) OR Pelvic Examinations) OR AI-Enabled Prototype OR Electronic) OR Follow-Up) OR Mobile-Application) OR Mobile-partograph) OR Partograph-Device) OR active labour) OR labour) OR cervix) OR partogram) OR partograph) or e-partogram or electronic-Touch OR mLabour) OR routine vaginal exam OR purple line OR uterine contraction OR OR 4D Transperineal Ultrasound) OR 4D Ultrasound) OR 4D) OR 3D Ultrasound) OR 3DUltrasound) OR 3D) OR Obstetric examination) OR electrohysterography OR electrohysterogram OR uterine electromyography OR uterine monitoring OR external tocodynamometer OR transperineal ultraso OR transperineal sonog OR transabdominal ultraso OR transabdominal sonog) AND (labour progress OR cervical dilatation studies published between 2000 and 2025 in English). This is used to obtain recent evidence on the digital devices/technologies used to measure vaginal examination and accurately estimate the progress of labour.
