# Supplementary material for: Mapping Intrusive and Non-Intrusive Ultrasound Technologies and Devices Used in Vaginal Examinations During Intrapartum: A Scoping Review
Source: Healthcare (Basel). 2026 Aug 3;14(15):2359. doi: 10.3390/healthcare14152359 (PMC13464107; doi:10.3390/healthcare14152359)
Supplement: Supplementary file 1 [file healthcare-14-02359-s001.zip › Search supplimentery (1).pdf]

Search	Add to builder	Query	Items found	Time
#76	Add	Search **((((((((((((((((((((((((((((((((((((((Devices for Measuring) AND examinations) AND digital vaginal) OR digital) AND vaginal) OR vaginal examination) OR Artificial Assisted intelligence) OR AI) OR Electronic) OR Follow-Up) OR Mobile-Application) OR Mobile-partograph) OR Partograph-Device) OR active labor) OR labor) OR cervix) OR partogram) OR partograph) OR effacement) OR mLabour) OR monitoring) OR progress) OR technique) OR ultrasound) OR 4D Transperineal Ultrasound) OR 4D Ultrasound) OR 4D) OR 3D Ultrasound) OR 3DUltrasound) OR 3D) OR Obstetric examination) OR Obstetric) OR electrohysterography) OR electro hysterogram) OR electro hysterography) OR transabdominal) OR labor progress) OR Pelvic Examinations) OR AI-Enabled Prototype**	772	02:15:51
#75	Add	Search **((((((((Devices for Measuring) AND Devices) AND Devices) AND Devices) AND Devices) AND Devices) AND Devices for Measuring) AND Devices for Measuring) AND Devices**	0	02:10:15
#74	Add	Search **AI-Enabled**	1	02:06:23
#73	Add	Search **AI-Enabled Prototype**	0	02:06:02
#72	Add	Search **Non-Intrusive**	0	02:05:49
#71	Add	Search **Non-Intrusive AI-Enabled Prototype for Vaginal Examinations**	0	02:05:39
#70	Add	Search **Pelvic Examinations**	1	02:04:22
#69	Add	Search **descent**	28	02:03:28
#68	Add	Search **fetal descent**	0	02:03:18
#67	Add	Search **'labor progress'**	0	02:02:55
#66	Add	Search **labor progress**	0	02:02:38
#65	Add	Search **transabdominal**	1	02:01:51
#64	Add	Search **sonog**	0	02:01:20
#63	Add	Search **trans perineal sonog**	0	02:01:08
#62	Add	Search **trans perineal**	0	02:00:46

Search	Add to builder	Query	Items found	Time
#61	Add	Search **trans perineal ultraso**	0	02:00:37
#60	Add	Search **tocodynamometer**	0	02:00:01
#59	Add	Search **uterine monitoring**	1	01:57:49
#58	Add	Search **electromyography**	2	01:56:55
#57	Add	Search **electro hysterography**	2	01:56:21
#56	Add	Search **electro hysterogram**	2	01:55:15
#55	Add	Search **hysterography**	0	01:54:42
#54	Add	Search **'electrohysterography'**	0	01:54:18
#53	Add	Search **electrohysterography**	0	01:54:00
#52	Add	Search **uterine contraction**	1	01:53:06
#51	Add	Search **OR uterine contraction**	1	01:52:56
#50	Add	Search **Obstetric**	44	01:51:15
#49	Add	Search **Obstetric examination**	0	01:50:56
#48	Add	Search **Cervixmetry**	0	01:50:21
#47	Add	Search **Cervimetry**	0	01:49:55
#46	Add	Search **3D**	156	01:45:24
#45	Add	Search **3DUltrasound**	0	01:45:15
#44	Add	Search **3D Ultrasound**	0	01:45:07
#43	Add	Search **4D**	28	01:44:13
#42	Add	Search **4D Ultrasound**	0	01:44:00
#41	Add	Search **4D Transperineal Ultrasound**	0	01:43:48
#9	Add	Search **ultrasound**	17	01:43:11
#40	Add	Search **technique**	156	01:42:06
#39	Add	Search **progress**	49	01:39:28
#38	Add	Search **monitoring**	100	01:38:16

Search	Add to builder	Query	Items found	Time
#37	Add	Search **mLabour**	0	01:37:58
#36	Add	Search **Birth**	38	01:37:01
#35	Add	Search **effacement**	7	01:36:24
#34	Add	Search **'electronic touch'**	0	01:35:28
#33	Add	Search **'electronic-Touch'**	0	01:35:15
#32	Add	Search **electronic-Touch**	0	01:34:47
#31	Add	Search **partograph**	0	01:34:25
#30	Add	Search **partogram**	0	01:33:51
#29	Add	Search **e-partogram**	7590	01:33:16
#28	Add	Search **cervix**	14	01:32:03
#27	Add	Search **centimetre**	0	01:31:36
#26	Add	Search **labor**	21	01:30:18
#25	Add	Search **active labor**	0	01:30:01
#24	Add	Search **accurately**	7	01:29:09
#23	Add	Search **Partograph-Device**	64	01:27:49
#22	Add	Search **Mobile-partograph**	65	01:25:43
#21	Add	Search **Mobile-Application**	1	01:24:38
#20	Add	Search **Follow-Up**	18	01:16:59
#19	Add	Search **Electronic**	21	01:13:35
#18	Add	Search **AI**	43	01:09:52
#17	Add	Search **Artificial Assisted intelligence**	0	01:09:36
#16	Add	Search **Artificial**	215	01:08:31
#15	Add	Search **VE**	19	01:07:52
#14	Add	Search **PV**	45	01:07:32
#13	Add	Search **vaginal examination**	1	01:06:41

Search	Add to builder	Query	Items found	Time
#12	Add	Search **vaginal**	31	01:05:42
#11	Add	Search **digital**	62	01:04:29
#10	Add	Search **digital vaginal**	0	01:04:18
#8	Add	Search **Reliability**	6	01:01:43
#7	Add	Search **Accurate**	17	01:00:34
#6	Add	Search **investigation**	23	00:57:57
#5	Add	Search **examinations**	18	00:56:18
#4	Add	Search **Cervical Dilation**	1	00:53:59
#3	Add	Search **Measuring**	81	00:51:41
#1	Add	Search **Devices**	51	00:49:06
#2	Add	Search **Devices for Measuring**	0	00:48:56
